# OA05.03. Impact of a mind-body medicine skills course on medical students’ perceived stress, mindfulness and elements of emotional intelligence

**DOI:** 10.1186/1472-6882-12-S1-O19

**Published:** 2012-06-12

**Authors:** K Motz, K Graves, C Gross, P Saunders, H Amri, N Harazduk, A Haramati

**Affiliations:** 1 Georgetown University School of Medicine, Washington D.C., USA

## Purpose

Most medical schools list self-awareness, self-care and personal growth as key educational competencies. Yet, studies have reported that traits such as altruism and empathy tend to decline throughout medical school training. To foster medical student self-awareness and mindfulness, which may improve their emotional intelligence (EI), Georgetown University School of Medicine (GUSOM) offers an experiential course to medical students in mind-body medicine skills. The purpose is to expose students to a variety of mind-body approaches (e.g., mindfulness meditation, autogenic training, guided imageries, movement, and writing exercises), as well as group sharing that involves listening generously and without judgment.

## Methods

The aim of this study was to assess the impact of the Mind Body Medicine Skills (MBS) course on several behavioral measures such as perceived stress (PSS), mindfulness (Freiberg Mindfulness Inventory – FMI), positive and negative affect (PANAS) and elements of EI (measured by the Trait Meta Mood Scale and the Interpersonal Reactivity Index).

## Results

Data were obtained from 72 first year medical students (47 females and 25 males), who completed the survey instruments before and after participating in the course. A significant decline was seen in perceived stress among all participants (17.4 vs. 13.3, p<0.001, effect size 0.73), while mindfulness increased significantly (35.3 vs. 40.0, p<0.001; effect size 0.94). Both males and females showed a significant increase in positive affect, but only females had a decrease in negative affect (males showed no change). Female students reported greater increases in attention to feelings and perspective taking, and a decrease in the personal distress in response to distress in others. However, both genders reported significant increases in empathic concern.

## Conclusion

These findings suggest that participation in a one-semester MBS course during the first year of medical school is effective in enhancing important traits such as mindfulness and empathic concern, while reducing students’ perceived stress.

